# DIP2B Interacts With α-Tubulin to Regulate Axon Outgrowth

**DOI:** 10.3389/fncel.2020.00029

**Published:** 2020-02-19

**Authors:** Zhen-Kai Xing, Lu-Qing Zhang, Yu Zhang, Xue Sun, Xiao-Lin Sun, Hua-Li Yu, Yao-Wu Zheng, Zi-Xuan He, Xiao-Juan Zhu

**Affiliations:** Key Laboratory of Molecular Epigenetics Ministry of Education, Institute of Genetics and Cytology, Northeast Normal University, Changchun, China

**Keywords:** DIP2B, axon guidance, axon outgrowth, tubulin, neuronal morphogenesis

## Abstract

Axonal development is essential to the establishment of neuronal morphology and circuitry, although the mechanisms underlying axonal outgrowth during the early developmental stages remain unclear. Here, we showed that the conserved disco-interacting protein B (DIP2B) which consists of a DMAP1 domain and a crotonobetaine/carnitine CoA ligase (Caic) domain, is highly expressed in the excitatory neurons of the hippocampus. DIP2B knockout led to excessive axonal outgrowth but not polarity at an early developmental stage. Furthermore, the loss of DIP2B inhibited synaptic transmission for both spontaneous and rapid release in cultured hippocampal neurons. Interestingly, DIP2B function during axonal outgrowth requires tubulin acetylation. These findings reveal a new conserved regulator of neuronal morphology and provide a novel intervention mechanism for neurocognitive disorders.

## Introduction

During neuronal development, neurons project axons and dendrites through cytoskeletal modulation reaching their cognate targets to establish neuronal networks (Hoogenraad and Bradke, 2009; Gallo, 2011; Kalil and Dent, 2014).To initiate neuronal morphogenesis, newborn neurons extend multiple neurites with a single neurite elongating and developing into axons, while the others extend slowly and differentiate into dendrites (Dotti et al., 1988; Goslin and Banker, 1989). This process is complex and dynamic and involves multiple signaling cascades to guide cytoskeletal remodeling (Barnes and Polleux, 2009; O’Donnell et al., 2009; Bashaw and Klein, 2010). A single neuron connects with divergent brain regions through its axons and these connections are critical to the formation of neural circuitry in the brain. To elucidate the molecular mechanisms underlying axonal development in establishing neural circuitry, numerous environmental factors and intracellular signaling pathways have been investigated (Poulain and Sobel, 2010; Lewis et al., 2013) but the mechanisms underlying axon development in the mammalian central nervous system remain unclear.

The disco-interacting protein 2 (DIP2) family was identified in *Drosophila* as a binding partner of *disconnected* and is conserved from *Caenorhabditis elegans* to humans (Mukhopadhyay et al., 2002). DIP2 contains a DNA methyltransferase- associated protein 1 (DMAP1) binding domain and Acyl-CoA synthetase (AMP-forming; Caic) domain, as well as AMP-binding sites. In *Caenorhabditis elegans*, DIP2 is critical to axonal regeneration in mature neurons (Noblett et al., 2019). Moreover, DIP2 regulates axonal bifurcation of mushroom body neurons in *Drosophila* (Nitta et al., 2017). Mammals express three *DIP2* genes (*DIP2A, DIP2B, and DIP2C*) each of which shares highly conserved functional domains and have similar roles in acetyl-coenzyme (acetyl-CoA) synthesis (Ma et al., 2019). In adult mice, DIP2A is expressed in multiple brain regions including the neocortex, hippocampus, amygdala, and cerebellum (Zhang et al., 2015). In humans, *DIP2A* is located on chromosome 21q22.3 and is associated with developmental delay (Yu et al., 2010). DIP2B plays an important role in DMA methylation in mitotic fetal epithelial progenitor cells during organogenesis (Hayashi et al., 2017). In humans, DIP2B is associated with the fragile site FRA12A on chromosome 12q13.1 and mediates neurocognitive disorders (Winnepenninckx et al., 2007). However, the functional role of the DIP2 family in mammals remains poorly understood.

To clarify this role, we investigated the expression patterns of DIP2B in adult mouse brains expressing a *LacZ* reporter. We found that DIP2B is highly expressed in hippocampal excitatory pyramidal neurons and its silencing facilitates axon development for both outgrowth and branching, decreasing dendrite length. DIP2B is critical for synaptic transmission in cultured hippocampal neurons. Furthermore, DIP2B interacts with α-tubulin, which is required for axon outgrowth. These results reveal a critical role for DIP2B in regulating axonal outgrowth and synaptic transmission, providing novel interventions for DIP2B related diseases in humans.

## Materials and Methods

### Animals

All procedures were approved by the Institutional Animal Care and Use Committee of Northeast Normal University (NENU/IACUC, AP2013011). The standard of the laboratory condition was specific pathogen-free (SPF). All the mice were group-housed (maximum four mice per cage) under a 12–12 h light-dark cycle (lights were on from 6:00–18:00 every day) with food and water provided *ad libitum*. C57BL/6J mice were from the Vital River (Beijing, China). *DIP2B* KO-first mice came from IMPC (MGI No. 2145977). FLP1 mice were obtained from Nanjing Biomedical Research Institute of Nanjing University (Nanjing, China). *NEX*-Cre mice were kindly provided by Prof. Zilong Qiu from the Chinese Academy of Sciences.

### Plasmids

The expression constructs encoding DIP2B have been maintained in our laboratory. The sequences for the shRNAs targeting the mouse DIP2B were as follows: *DIP2B* shRNA3026: 5′-GCTGCCTTCAGCTTCATAAGC-3′; *DIP2B* shRNA3959: 5′- GGATCAATCTTTCTTGCATCC-3′. The DIP2B shRNAs were cloned into the PLL 3.7 vector. The cDNA encoding DIP2B different truncations (1–333 aa, 334–992 aa, and 993–1574 aa) were amplified by PCR and cloned into the pEGFP-C1 vector and the PEX-5X-1 vector respectively. Tubulin and TubulinK40Q were purchased from Addgene or kindly provided by Tso-Pang Yao from Duke University.

### Antibodies

Primary antibodies used were: rabbit anti-DIP2B (1:2,000; Sigma HPA046133), mouse anti-α tubulin (1:5,000; Abcam ab7291), mouse anti-ace α tubulin (1:5,000; Sigma T7451), chicken anti-LacZ (1:2,000; Aves AB_2313508), mouse anti-tau1 (1:1,000; Abcam ab75714), rabbit anti-CamKII (1:500; Abcam ab52476), mouse anti-GABA (1:500; Sigma A0310), mouse anti-NeuN (1:800; Millipore MAB377), rabbit anti-GFAP (1:500; Abcam ab7260), chicken anti-GFP (1;2,000; Aves AB_2307313), mouse anti-GAPDH (1:5,000; Transgen HC301-01), rabbit anti-GST (1:2,000; CST 2625), mouse anti-His (1:2,000; Abcam ab18184).

Secondary antibodies used were: anti-Mouse IgG-HRP (1:5,000; Santa Cruz Biotechnology sc-2005), Goat-anti-Rabbit IgG-HRP (1:5,000; Invitrogen PI31460), Donkey anti-Mouse Alexa Fluor 488 (1:5,000; Invitrogen A21202), Goat anti-Chicken Alexa Fluor 488 (1:5,000, Invitrogen A11039), Donkey anti-Rabbit Alexa Fluor 546 (1:5,000, Invitrogen A10040), Donkey anti-Mouse Alexa Fluor 546 (1:5,000, Invitrogen A10036).

### Genotyping

Genomic DNA was extracted from tail tips. Samples were treated with Mouse Direct PCR Kits. For *LacZ* insertion, the following primers were used: Forward primer: 5′-ACCACACCTCCTGCTGTATAC-3′ and Reverse primer: 5′-ACGACGGGATCATCGCGAGCCAT-3′ (Zhang et al., 2015). For *LacZ* homozygote and heterozygote identification, the following primers were used: Forward primer: 5′-AGTTAAGGCTGAGCATGGTGGGA-3′ and Reverse primer: 5′-TAGGGCTCTCACAGATCAGAGCT-3′. For *NEX*-cre; *DIP2B*^floxp/floxp^ genotyping, the following primers were used: Forward primers: 5′-CCGCATAACCAGTGAAACAG-3′, Reverse primers: 5′-AGAATGTGGAGTAGGGTGAC-3′ and Reverse primers: 5′-GAGTCCTGGCAGTCTTTTTC-3′.

PCR reactions were running using the following parameters: initial denaturation at 94°C for 2 min, 30 cycles of 94°C for 30 s, 57°C for 30 s and 72°C for 30 s, followed by a final extension of 72°C for 5 min with a little bit modification of anneal temperature which is dependent on specificity of primers.

### Western Blotting

Western blotting procedures were performed as previously described (He et al., 2019). The protein samples were fractionated using SDS-PAGE and transferred to PVDF membranes (Millipore). Membranes were blocked in 5% non-fat milk in TBST at room temperature for 60 min and probed with primary antibodies diluted in 2% BSA in TBST at 4°C overnight. Membranes were then washed in TBST and labeled with 1:5,000 HRP-conjugated secondary antibodies (Sigma) for 1 h. Protein bands were detected with Tanon High-sig ECL Western blotting Substrate (Tanon, Shanghai, China, Cat. No. 180-5001). The blots were imaged with high definition and low illumination CCD system (Tanon 5,500). The quantification for the results was carried on Fiji/ImageJ.

### LacZ Staining

Adult mice were anesthetized with 0.7% pentobarbital (10 mg/kg) and perfused with 4% paraformaldehyde. Tissues were harvested and postfixed in 4% paraformaldehyde for 2 h at 4°C, washed with LacZ staining buffer, and immersed in 35% sucrose (by volume) for 72 h at 4°C. The sections were frozen at optimal cutting temperatures for sectioning using a cryostat. The samples were washed in rinse buffer (2 mM MgCl_2_, 0.02% NP40 and 0.01% Na-deoxycholate in PBS) and stained with 30 mM K_3_Fe(CN)_6_, 30 mM K_4_Fe(CN)_6_3H_2_O, 2 mM MgCl_2_, 0.01% Na-deoxycholate, 0.02% NP40 and 1 mg/ml 5-Bromo-4-chloro-3-indolyl-β-D-galactopyranoside (X-Gal; Sigma) in PBS at 37°C for 6–12 h. Embryos were washed in PBS, post-fixed overnight in 4% PFA with agitation at 4°C, and washed. Stained tissues and embryos were stored in 70% glycerol at 4°C and imaged with an Olympus microscope (FSX100, Japan).

### Immunohistochemistry

Brains were fixed in 4% PFA overnight at 4°C and placed in 35% sucrose in PBS for 2 days. Brains were sectioned on a freezing microtome (Leica, CM 1950) at 40 μm. Frozen sections were washed with PBS and antigen retrieval was performed in 0.01 M sodium citrate buffer (pH 6.0) at 98°C for 5 min. The sections were blocked with 2% BSA in 0.2% Triton X-100/PBS for 1 h, and slices were incubated with primary antibodies overnight at 4°C. After washing five times with 0.1% Tween-20 in PBS, the slices were incubated with the appropriate fluorochrome-conjugated secondary antibodies for 1 h. The sections were mounted and sealed with PVA. Images were captured on an Olympus FSX100 microscope with a 60× UPLSAPO objective (NA = 1.35).

### Primary Hippocampal Neuronal Cultures

Hippocampus from P0 mice was dissected in Hank’s balanced salt solution (HBSS), and digested with 0.15% trypsin at 37°C for 20 min and dissociated with a 1 ml pipette. Neurons were plated into Dulbecco’s modified eagle’s medium mixture F12 (Sigma; DMEM-F12) containing 10% fetal bovine serum on 18 mm glass bottoms coated dishes with poly-l-lysine (50 μg/ml sigma). Neurons attached to the substrate were incubated with nerobasalA medium containing 2% B-27 supplements and 2 mM L-Glutamax (ThermoFisher).

### Time-Lapse Imagining

Hippocampal neurons were cultured on glass-bottom cell culture dishes for 4 h and placed on a temperature-controlled workstation (37°C, 5% CO_2_) with an inverted microscope (PerkinElmer Ultraview Vox). To observe axonal growth and axonal branching, primary neurons were imaged at 10 min intervals for 40 h (20× magnification).

### Whole-Cell Recording

All recordings were performed at room temperature with recording solution containing 120 mM NaCl, 2.5 mM KCl, 1.0 mM NaH_2_PO_4_, 26 mM NaHCO_3_, 11 mM D-glucose, 2.0 mM MgCl_2_, and 2.0 mM CaCl_2_ (pH 7.4, with osmolarity of 295—315 mOsm). Whole-cell patch-clamp recordings of cultured hippocampal neurons were performed in the voltage-clamp mode using an EPC-10/2 amplifier (HEKA, Germany). Recording pipettes were pulled from borosilicate glass tubes (Sutter Instruments) at a resistance of 3–6 MΩ; and membrane potentials were held at −70 mV. The pipette solution consisted of 130 mM K-gluconate, 1 mM EGTA, 5 mM Na-phosphocreatine, 2 mM Mg-ATP, 0.3 mM Na-GTP, 5 mM QX-314 (TOCRIS Bioscience) and 10 mM HEPES, pH 7.4.

For mEPSC recordings, cultured hippocampal neurons were held at −70 mV under voltage-clamp. Tetrodotoxin (1 μM; TOCRIS Bioscience), 50 μM D-AP5 (TOCRIS Bioscience) and 20 μM bicuculline (TOCRIS Bioscience) were added to the bath solution.

To measure evoked EPSC, cultured hippocampal neurons were recorded in the presence of 50 μM D-AP5 and 20 μM bicuculline. Presynaptic inputs were stimulated with a CBAEC75 concentric bipolar electrode (FHC, USA) which was placed near (~100–150 (μm). Data were acquired using PATCHMASTER software (HEKA, Germany) and analyzed using MiniAnalysis software (Synaptosoft), Clampfit (Molecular Devices), and Igor (Wavemetrics).

### GST Pulldowns

GST- DIP2B-Caic or GST recombinant proteins immobilized on GSH-agarose were incubated overnight with wild type (WT) P56 mouse brain extracts. Beads were extensively washed and eluted proteins run on SDS-PAGE. Proteins were stained with Coomassie Brilliant Blue G-250. Protein bands from GST-DIP2B-Caic were identified by mass spectrometry.

### Immunoprecipitation

HEK293T cells were transfected with calcium phosphate. Cell lysates were mixed with 1 mg of antibody overnight at 4°C, followed by incubation with Protein A/G-agarose beads (Roche) for 2 h at 4°C. After thorough washing, samples were boiled and analyzed by Western blotting using standard protocols.

### Statistical Analysis

Data were acquired from at least three independent experiments and are presented as the mean ± SEM. Data were compared using a student’s *t*-test or a one-way ANOVA (Prism software). *P-values < 0.05* were deemed statistically significant.

## Results

### DIP2B Expression in the Developmental Brain

**Figure 1:**
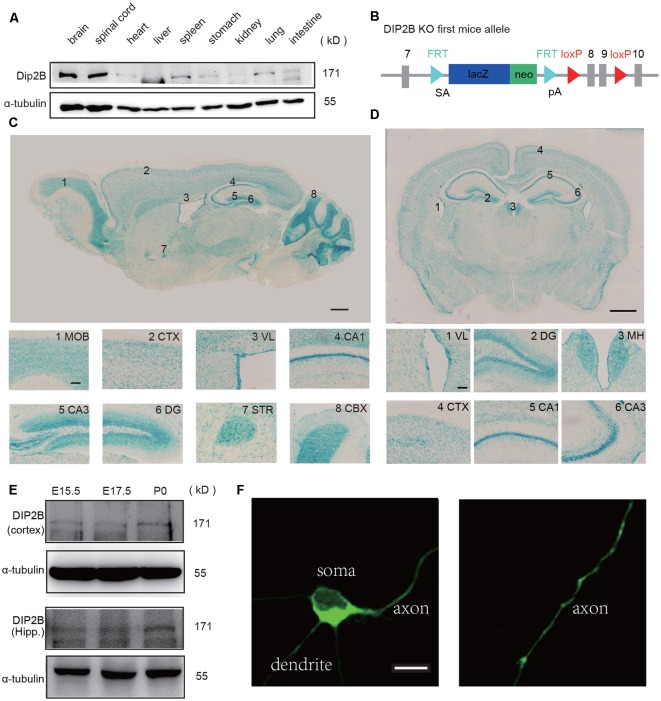
DIP2B is highly expressed in excitatory hippocampal neurons. **(A)** Western blots of DIP2B expression in the different mouse organs. **(B)** Schematic depiction of DIP2B-LacZ mice. **(C)** Staining of sagittal slices from DIP2B-LacZ mice. Scale bar: 1 mm; subfigure: 50 μm. **(D)** Staining of coronal slices from DIP2B-LacZ mice. Scale bar: 1 mm; subfigure: 50 μm. **(E)** DIP2B expression during early developmental stages. **(F)** Overexpression of DIP2B-GFP in cultured hippocampal neurons. Scale bar: 10 μm.

The expression of DIP2B was assessed in multiple tissues by western blotting analysis. We found that DIP2B was highly expressed in the brain and spinal cord (Figure 1A) indicating an important role in the nervous system. *LacZ* reporters were inserted into the murine *DIP2b* genome between exon 7 and 8 to assess DIP2B expression patterns in the brain (Figure 1B). Staining of sagittal slices (Figure 1C) revealed that DIP2B is expressed in the main olfactory bulb (MOB), cortex (CTX), lateral ventricle (VL), cornu ammonis1 (CA1), cornu ammonis3 (CA3), dentate gyrus (DG), striatum (STR), cerebellar cortex (CBX) and medial habenula (MH). The staining of coronal slices showed similar results (Figure 1D). From immunohistochemistry analysis, DIP2B was expressed mostly in neurons, including excitatory pyramidal neurons (95.89 ± 1.3%) and inhibitory interneurons (79.43 ± 1.7%; Supplementary Figures S1A–F). Furthermore, the expression of DIP2B was initiated at the embryonic stage in E15.5 in both the neocortex and hippocampus indicating that DIP2B regulates neuronal development (Figure 1E). Giving that commercial antibody of DIP2B did not work well for the immunostaining, to investigate the expression pattern of DIP2B at the cellular level, we over-expressed the DIP2B construct in cultured hippocampal neurons and found that DIP2B overexpressed protein was distributed all over the cell including soma, dendrites, and axon (Figure 1F).

### DIP2B Regulates Axonal Morphogenesis

**Figure 2:**
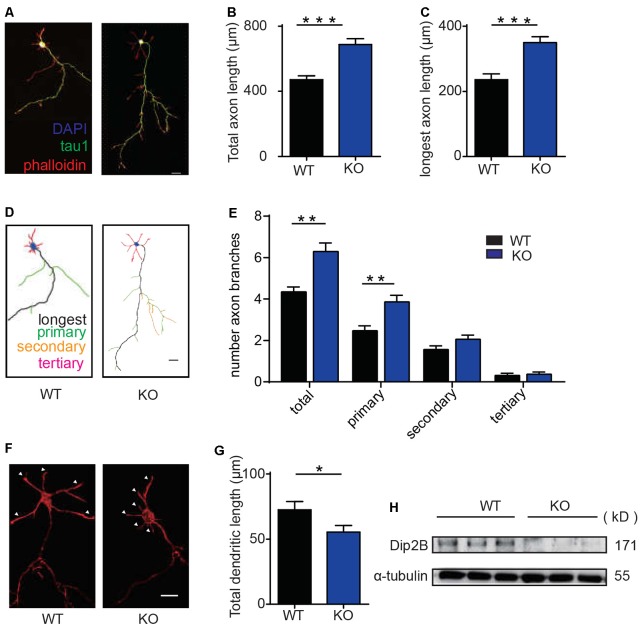
DIP2B is required for axon development in cultured hippocampal neurons. **(A)** Representative imagines of axonal development at DIV4 from cultured hippocampal neurons. wild type (WT): littermate controls of DIP2B KO mice; KO: DIP2B knockout mice. Green: tau-1; Red: phalloidin; Blue: DAPI. Scale bar: 20 μm (WT, *n* = 40; KO, *n* = 51). **(B)** Average total axonal length from panel **(A)**. **(C)** The average length of the longest axon from panel **(A)**. **(D)** Schematic depiction of axonal development at DIV4 from cultured hippocampal neurons. Scale bar: 20 μm. **(E)** Average axonal branching from panel **(A)**. **(F)** Representative imagines of dendritic outgrowth at DIV4 from cultured hippocampal neurons. WT: littermate controls of DIP2B KO mice; KO: DIP2B knockout mice. Red; phalloidin. Scale bar: 20 μm. **(G)** Average dendritic length. **(H)** DIP2B expression in the brains of control and DIP2B KO mice. An unpaired *t-*test was used for comparisons. *p < 0.05; **p < 0.01; ***p < 0.001; n, cell number. Error bars represent SEM. For further details, see Supplementary Table S1.

Recent studies in flies and worms indicate that DIP2 is required for axonal guidance during early developmental stages and regeneration in mature neurons (Nitta et al., 2017; Noblett et al., 2019). To determine the role of DIP2B in neurite outgrowth, we quantified axonal outgrowth and branching in cultured hippocampal neurons. Hippocampal neurons from P0 mice were cultured *in vitro* for 4 days and imaged for the comparison of axonal morphology between WT and DIP2B knockout mice. We found that DIP2B knockout increased the total axon length and the length of the longest axon with Tau1 staining (Figures 2A–C). Furthermore, DIP2B knockout enhanced the number of primary axon branches (Figures 2D,E). However, not all neurites developed abundant outgrowth, at day 4 *in vitro* (DIV), and the lengths of dendrites from DIP2 knockout mice significantly decreased compared to control mice (Figures 2F,G). These results were confirmed by western blotting (Figure 2H). Taken together, DIP2B was identified as an important regulator for neurite outgrowth and branching during neuronal development, suggesting that there are distinct mechanisms underlying the differential growth of axons and dendrites (Wang et al., 2014).

### DIP2B Knockdown Enhances Axonal Outgrowth

**Figure 3:**
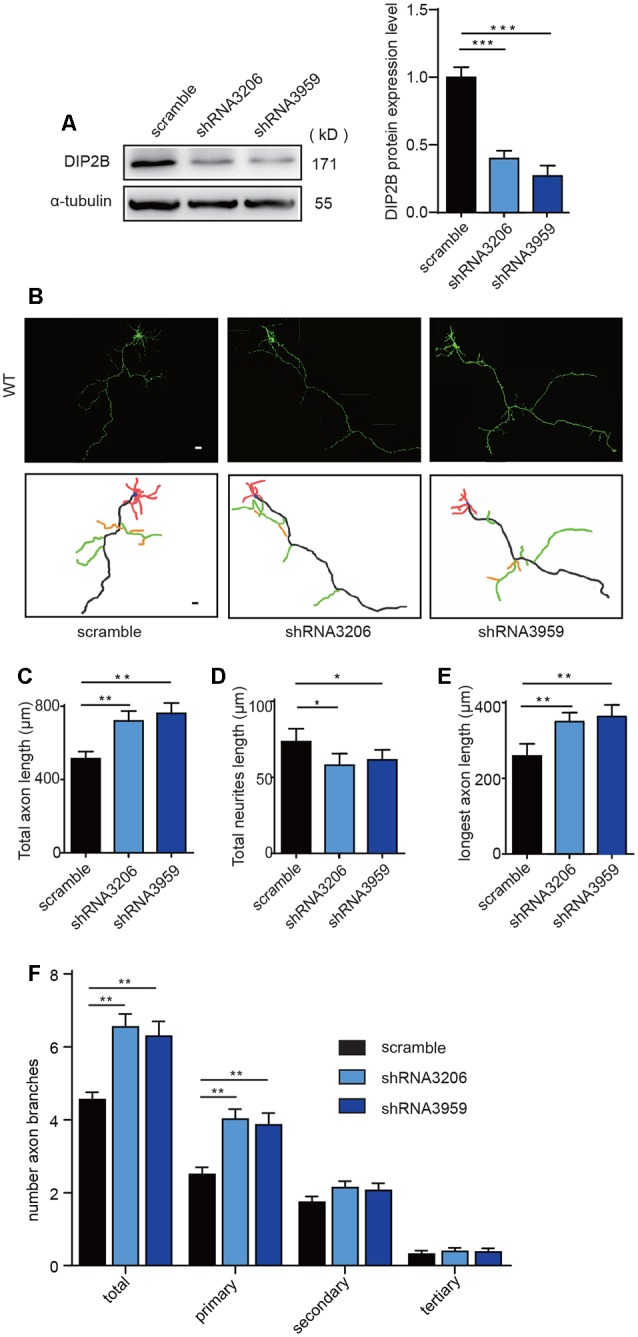
DIP2B silencing enhances axon outgrowth. **(A)** Western blotting confirmed the knockdown efficiency of DIP2B shRNA 3206 and shRNA 3959 through three independent experiments. **(B)** Representative images of axonal development at DIV4 from cultured hippocampal neurons transfected with scrambled, shRNA 3206 and shRNA 3959. Scale bar: 20 μm (Scramble, *n* = 31; shRNA 3,206, *n* = 36; shRNA 959, *n* = 36). **(C)** Average total axonal length from panel **(B)**. **(D)** Average total neurites length from panel **(B)**. **(E)** The average length of the longest axon from panel **(B)**. **(F)** Average axon branching from panel **(B)**. One-way ANOVA was used for the comparison. *p < 0.05; **p < 0.01; ***p < 0.001; n, cell number. Error bars represent SEM. For further details, see Supplementary Table S1.

The effects of DIP2B silencing on axonal and dendritic morphogenesis were further verified. We constructed DIP2B-shRNA lentiviruses (3206 and 3959) targeting murine *DIP2B* and infected wild-type mouse neurons. Knockdown efficiency was confirmed by Western blotting from cultured neurons transfected with shRNA-3206 and shRNA-3959 (Figure 3A). We compared cultured hippocampal neurons transfected with shRNA constructs (3206 and 3959) and in neurons transfected with scrambled shRNA. We found that knockdown of DIP2B in cultured hippocampal neurons increased axonal length and branching, but decreased dendritic outgrowth at DIV4 (Figures 3B–F). These findings were consistent with the results from DIP2B knockout mice and indicated that DIP2B regulates axonal outgrowth during early developmental stages.

### DIP2B Regulates Axon Outgrowth Behind Polarity Formation

To study whether DIP2B silencing facilitates axonal polarity or promotes axonal outgrowth, we performed time-lapse imaging to monitor the dynamic processes of axon initiation in cultured hippocampal neurons during the early developmental stage. During the first 24 h post-plating, culture neurons formed lamellipodia around the cell body and developed minor neurites (Dotti et al., 1988). The hallmark of this process was that a single neurite began to grow quickly to become the axon, referred to as axon polarity. This was a critical stage in initiating breaks in symmetry during neuronal development (Craig and Banker, 1994).

**Figure 4:**
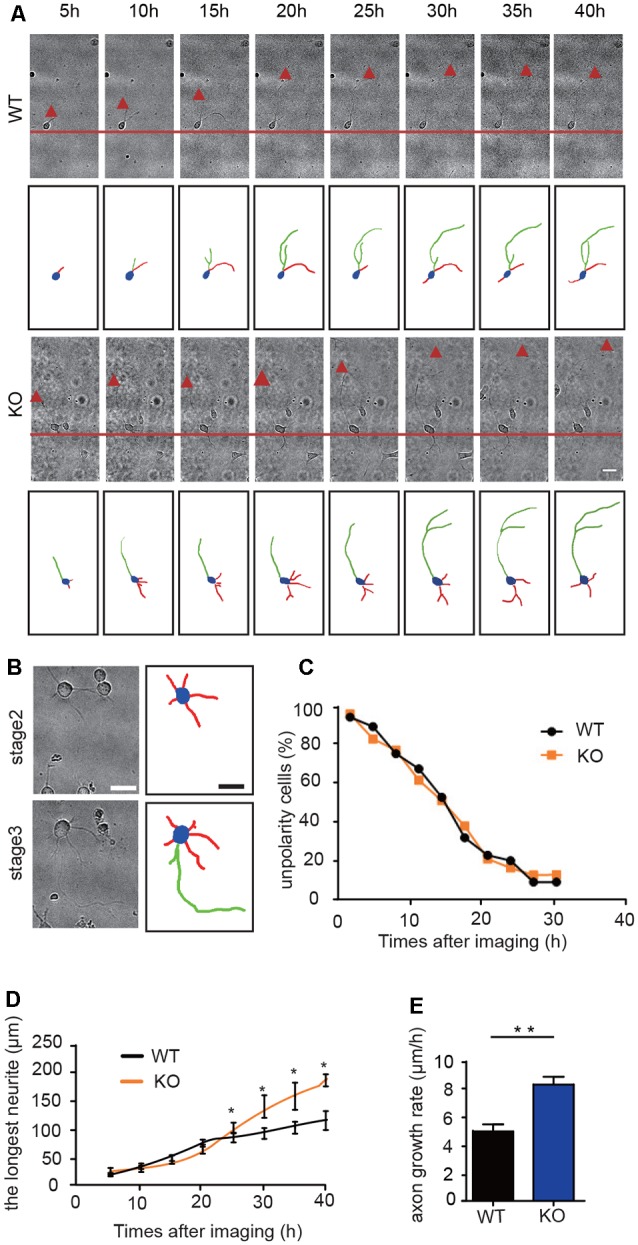
DIP2B is critical for axon outgrowth following polarity formation. **(A)** Representative images of axonal development during the first 40 h of plating. WT: littermate controls of DIP2B knockout mice; KO: DIP2B knockout mice. Scale bar: 20 μm. **(B)** Representative images of stage 2 and 3 axonal development. Scale bar: 40 μm. **(C)** The average percentage of unpolarized to total neurons. **(D)** Average traces of neurite outgrowth from 20 to 40 h post-plating (WT, *n* = 83; KO, *n* = 82). **(E)** Average axonal growth rates from panel **(D)**. An unpaired *t-*test was used for comparisons. *p < 0.05; **p < 0.01; n, cell number. Error bars represent SEM. For further details, see Supplementary Table S1.

We next assessed the stages of axon initiation including axon polarity establishment and outgrowth using cultured hippocampal neurons (Figure 4A). During the first 24 h of live cell observations, the neurons and neurites dynamically extended at stage 2 and established an axon at stage 3 (Figure 4B). The ratio of non-polarity to total neurons showed no significant differences between DIP2B KO and WT neurons (Kaech and Banker, 2006; Namba et al., 2014; Figures 4B,C). After 24 h, the axonal growth of DIP2B KO neurons increased compared to WT neurons (Figures 4D,E), suggesting that the abundant axonal development in DIP2B knockout neurons is due to the abnormalities of axon outgrowth rates. Taken together, DIP2B was identified as critical for axonal outgrowth during neuronal development.

Furthermore, we performed time-lapse experiments to clarify the role of DIP2B in axonal outgrowth using *DIP2B*^loxP/loxP^; *NEX-cre* mouse lines. Cultured hippocampal neurons were obtained from P0 *DIP2B*^floxp/floxp^ and *DIP2B*^floxp/floxp^; *NEX-cre* mice, *DIP2B*^floxp/floxp^ mice served as control. DIP2B knockout efficiency was verified by Western blot (Supplementary Figures S2A,B). Obviously, the deletion of *DIP2B* from excitability neurons specifically promoted axon outgrowth but not polarity (Supplementary Figures S2C–F). These results were confirmed by *DIP2B* knockout mice and indicated that DIP2B regulates axonal outgrowth during early developmental stages.

### DIP2B Is Required for Synaptic Transmission in Cultured Hippocampal Neurons

**Figure 5:**
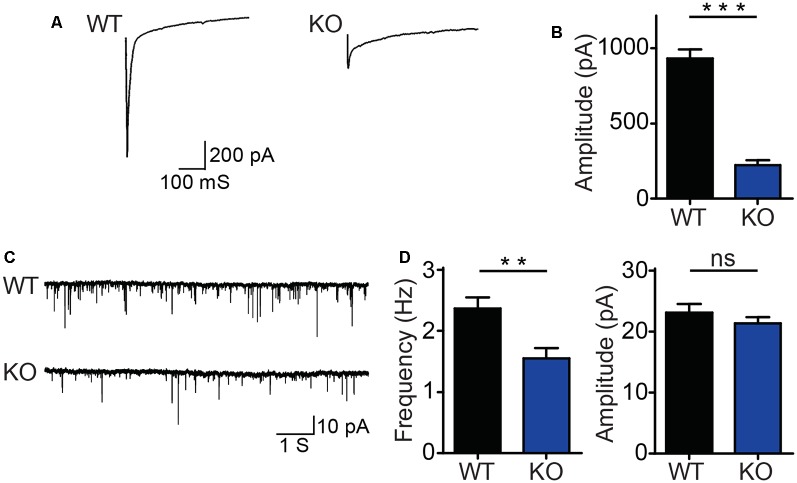
DIP2B knockout inhibits synaptic transmission in hippocampal neurons. **(A)** Representative recordings of excitatory postsynaptic currents from WT and KO hippocampal neurons (WT, *n* = 10; KO, *n* = 10). **(B)** The average amplitude of EPSC recorded from panel **(A)**. **(C)** Representative recordings of excitatory spontaneous release from WT and KO mice (WT, *n* = 13; KO, *n* = 14). **(D)** Average frequency (left) and amplitude (right) of mEPSC recorded. An unpaired *t-*test was used for comparisons. ns, not significant; **p < 0.01; ***p < 0.001; n, cell number. Error bars represent SEM. For further details, see Supplementary Table S1.

The morphology of neurons is essential for neurotransmitter release and synaptic function (Harris and Weinberg, 2012). To study the functional role of DIP2B in the brain, we examined whether DIP2B is critical for rapid synaptic transmission in cultured hippocampal neurons. Electrophysiological recordings of AMPAR-mediated evoke excitatory postsynaptic currents (EPSC) were performed using DIV15–17 cultured hippocampal neurons from *DIP2B* knockout and WT mice. DIP2B knockout neurons exhibited a 3-fold reduction in EPSC amplitude (Figures 5A,B), suggesting that DIP2B plays an important role in synaptic transmission. In the following experiments, AMPAR-mediated excitatory postsynaptic currents (mEPSC) were measured using DIV15–17 cultured hippocampal neurons from DIP2B knockout and WT mice. We found that DIP2B knockout significantly reduced mEPSC frequency in comparison to WT neurons, with no marked differences in mEPSC amplitude (Figures 5C,D). Previous work reported decreased mEPSC frequency is associated with reduced spine density (He et al., 2019). Giving that DIP2B knockout significantly inhibited the growth of dendrite, we propose that the dysfunction of morphogenesis may be critical for the defect of synaptic transmission in DIP2B knockout mice.

### DIP2B Regulates Axonal Outgrowth by Interaction With α-Tubulin

**Figure 6:**
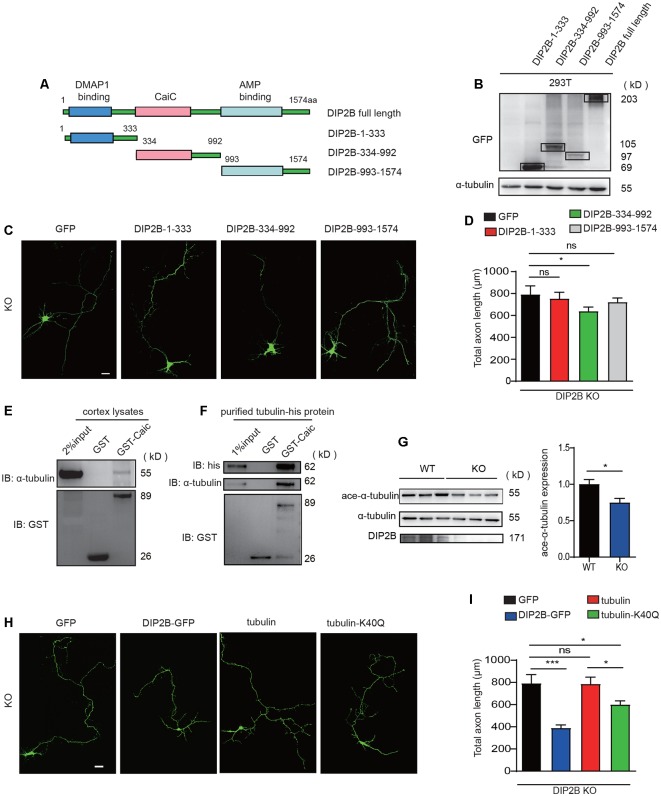
Acetylation of α-tubulin is critical for the regulation of DIP2B during axonal outgrowth. **(A)** A schematic image showing constructs for different DIP2B domains. **(B)** Immunoblot (IB) of protein extracts from 293T cells. **(C)** CaiC domain was critical for DIP2B regulating axon outgrowth (KO+GFP, *n* = 23; KO+DIP2B-1-333, *n* = 21; KO+DIP2B-334-992, *n* = 24; KO+DIP2B-993-1574, *n* = 23). Scale bar: 20 μm. **(D)** Averaged axon shown in **(C)**. **(E)** P56 cortical lysates were incubated with the GST-DIP2B-Caic and GST proteins as bait and then evaluated by Western blotting. **(F)** Purified tubulin was incubated with the GST-DIP2B-Caic and GST proteins as bait and then evaluated by Western blotting. **(G)** Immunoblot analysis exhibited that α-tubulin acetylation was decreased in DIP2B KO mice. *n* = 6, data came from two independent experiments. **(H)** K40Q is critical for DIP2B regulating axon outgrowth (WT, *n* = 15; KO, *n* = 16; KO+DIP2B, *n* = 22; KO + tubulin, *n* = 23; KO+K40Q, *n* = 22). Scale bar: 20 μm. **(I)** Average total axon length from panel **(H)**. One-way ANOVA was used for comparisons. ns, not significant; *p < 0.05, ***p < 0.001; n, cell number. Error bars represent SEM. For further details, see Supplementary Table S1.

To investigate the underlying mechanisms that DIP2B regulates axonal outgrowth, we first clarify which domain is crucial to the function of DIP2B. Truncation of DIP2B constructs that fused with enhanced GFP (eGFP) with domain features (Figures 6A,B) were transfected into cultured neurons at DIV1. We found that the CaiC domain (334–992 aa) inhibited axon outgrowth at DIV 4 *in vitro* (Supplementary Figures S3A,B). Importantly, the transfected DIP2B CaiC domain (334–992) reduced the length of the overgrown axon of *DIP2B* KO neurons (Figures 6C,D), implying DIP2B is critical for regulating axonal outgrowth in cultured neurons. To explore the regulatory mechanism of the DIP2B Caic domain in axonal outgrowth, we performed liquid chromatography-tandem mass spectrometry (LC-MS/MS) to identify binding partners from murine brain lysate that incubated with GST-CaiC. A total of 505 proteins were found and abundant proteins exhibited tubulin-binding activities (Supplementary Figure S3D). With co-transfection of tagged DIP2B and α-tubulin together, we explored DIP2B (Supplementary Figure S3C) interaction with tubulin by immunoprecipitation assays. As expected, over-expressed DIP2B and α-tubulin interacted with each other (Supplementary Figure S3E). The binding activity was further confirmed by GST pull-down assays with GST-CaiC in the murine brain lysate (Figure 6E), also purified tubulin bound with CaiC *in vitro* (Figure 6F). These results indicated that DIP2B interacted with tubulin directly.

Alpha-tubulin is important for axon guidance and outgrowth, acetylation of α-tubulin regulates the properties of microtubules. Thus, in the following experiments, we investigated whether DIP2B regulated axon outgrowth *via* mediating α-tubulin acetylation. First, we found that the knockout of DIP2B decreased the acetylation level of α-tubulin (Figure 6G). Next, acetylation mimetic α-tubulin by mutating K_40_ to Q_40_ (Li et al., 2012; Yu et al., 2015) was able to partially rescue the excessive outgrowth phenotype in DIP2B knockout neurons (Figures 6H,I). Above all, these results suggested that DIP2B may modulate axon outgrowth by regulating α-tubulin acetylation.

## Discussion

Genetic studies in humans implicate DIP2B is involved in neurocognitive disorders (Winnepenninckx et al., 2007). However, the function of DIP2B in the mammalian central nervous system remains unclear. In this study, we demonstrated that DIP2B knockout mice have abundant axonal development and synaptic transmission defects. The characteristics of these aberrations resemble the core symptoms of neurodevelopmental disorders. Our findings, therefore, strengthen the evidence that DIP2B is associated with neurocognitive disorders.

DIP2B contains a crotonobetaine/carnitine CoA ligase (Caic) domain that is related to acyl-CoA synthetases. The mechanism(s) by which acyl-CoA modulates axonal outgrowth and branching remains unclear. We found that DIP2B interacts with α-tubulin *in vitro*. The acetylation of α-tubulin was critical to the stabilization of microtubules and the dynamic nature of the microtubule cytoskeleton is critical for neuronal development. Recently, it was reported that α-tubulin acetylation restricts axonal over-branching by dampening microtubule plus-end dynamics (Dan et al., 2018). Our results indicate that DIP2B inhibits axonal outgrowth through its interaction with α-tubulin in hippocampal neurons. DIP2B may be relevant for the synthesis of acyl-CoA and is required for the acetylation of α-tubulin. Thus, the regulation of DIP2B may be essential to axonal development in the neurons at early developmental stages.

Neuronal polarity is established during early development. The most important step of polarity is the initiation of symmetry-breaking cues, such as extracellular signals or intracellular cytoplasmic factors that provide neurons with specific orientations (Yogev and Shen, 2017). Neuronal polarity is inherited from neuroepithelial progenitor cells (Morgan et al., 2006; Zolessi et al., 2006). Multiple signaling pathways are involved in axonal initiation and polarity (Polleux et al., 2002; Goldstein and Macara, 2007). Appropriate regulation of the actin and microtubule cytoskeleton is required for polarity. In unpolarized stage 2 hippocampal neurons, actin-based remodeling of the cytoskeleton in a single neurite is critical to conferring axonal polarity (Bradke and Dotti, 1999). We found that DIP2B regulates axonal outgrowth but not polarity during early developmental stages. These results indicate that DIP2B does not lead to actin cytoskeleton rearrangements during neuronal development.

Notably, DIP2B KO hippocampal neurons display synaptic transmission for both spontaneous and rapid release. Critically, our data provide a direct link between alterations of synaptic function and neuronal morphological abnormalities. The axonal and dendritic aberrations in neurons are accompanied by synaptic transmission defects (Südhof, 2018). Given that DIP2B is highly expressed during adult developmental stages, we were unable to clarify whether synaptic transmission defects are a direct result of neuronal morphological alterations. Nevertheless, our data provide a novel mechanism underlying axonal outgrowth and highlight new therapeutic targets for neurocognitive disorders.

In summary, we found that DIP2B is critical to axonal development in hippocampal neurons. During early developmental stages, DIP2B silencing promotes axonal development and inhibits dendrite outgrowth. DIP2B regulates axonal outgrowth but not polarity. DIP2B silencing results in synaptic transmission defects in hippocampal neurons. Furthermore, DIP2B regulates the acetylation of tubulin, which is critical for axonal outgrowth. The discovery that DIP2B regulates neuronal development and synaptic function may improve therapeutic strategies for neurocognitive disorders.

## Data Availability Statement

All datasets generated for this study are included in the article/Supplementary Material.

## Ethics Statement

The animal study was reviewed and approved by The Institutional Animal Care and Use Committee of Northeast Normal University.

## Author Contributions

Z-KX conducted all the experiments except electrophysiology experiments. Z-XH carried out the patchwork. L-QZ, YZ, XS, X-LS, H-LY, and Y-WZ helped with biochemistry experiments. Z-XH and X-JZ co-wrote the article.

## Conflict of Interest

The authors declare that the research was conducted in the absence of any commercial or financial relationships that could be construed as a potential conflict of interest.

## Supplementary Material

FIGURE S1 DIP2B expression at the cellular level. **(A)** Immunocytochemistry of the LacZ with NeuN; **(B)** LacZ with GFAP; **(C)** LacZ with CamKII; **(D)** LacZ with GABA. **(E)** Relative quantification of LacZ, NeuN, and GFAP positive cells. **(F)** Relative quantification of LacZ, CamKII, and GABA positive cells. Scale bar: 20 mm.

FIGURE S2 DIP2B is critical for axonal outgrowth in cKO mouse neurons. **(A,B)** DIP2B conditional knockout efficiency. Ctrl: DIP2Bfloxp/floxp, cKO: DIP2Bfloxp/floxp; NEX-Cre. **(C)** Representative images of axonal development during the first 40 h of plating from Ctrl and cKO mice. Scale bar: 20 mm. **(D)** The average percentage of unpolarized to total neurons. **(E)** Average traces of neuritis outgrowth during the first 40 h of plating (Ctrl, *n* = 40; cKO, *n* = 42). **(F)** Average axonal growth rates from panel **(D)**. An unpaired *t*-test was used for comparisons. **p* < 0.05; ***p* < 0.01; n, cell number. Error bars represent SEM. For further details, see Supplementary Table S1.

FIGURE S3 CaiC domain is critical for DIP2B regulating axon outgrow. **(A)** WT mice neurons transfected with DIP2B-1-333, DIP2B-334-992 and DIP2B-993-1574 constructs that fusion with enhanced GFP, respectively, then stained with the GFP antibodies at DIV 4 (WT+GFP, *n* = 25; WT+DIP2B-1-333, *n* = 24; WT+DIP2B-334-992, *n* = 22; WT+DIP2B-993–1574, *n* = 21) Scale bar: 20 mm. Quantitative analysis of the total axon length results shown in **(B)**. **(C)** Coomassie blue staining of purified GST-DIP2B-Caic shown in the red frame, which was semi quantitative with BSA loading. Protein bands for liquid chromatography-tandem mass spectrometry were exhibited in frame that pulls down from murine brain lysate. **(D)** GO biological process analysis of proteins identified from liquid chromatography-tandem mass spectrometry. **(E)** Microtubule related proteins found in LC-MS/MS. **(F)** Interaction of DIP2B and a-tubulin. HEK293T cells were co-transfected with pCDNA3.1DIP2B and a-tubulin-GFP. Cell lysates were immunoprecipitated with anti-myc and GFP antibody respectively. One-way ANOVA was used for comparisons. ns, non significant; **p* < 0.05; n, cell number. Error bars represent SEM. For further details, see Supplementary Table S1.

TABLE S1 Detail data analysis.
