# Supplementary material for: DIP2B Interacts With α-Tubulin to Regulate Axon Outgrowth
Source: Front Cell Neurosci. 2020 Feb 19;14:29. doi: 10.3389/fncel.2020.00029 (PMC7045754; doi:10.3389/fncel.2020.00029)
Supplement: FIGURE S1 — DIP2B expression at the cellular level. (A) Immunocytochemistry of the LacZ with NeuN; (B) LacZ with GFAP; (C) LacZ with CamKII; (D) LacZ with GABA. (E) Relative quantification of LacZ, NeuN, and GFAP positive cells. (F) Relative quantification of LacZ, CamKII, and GABA positive cells. Scale bar: 20 mm. [file Data_Sheet_1.pdf]

Supplementary Figure1

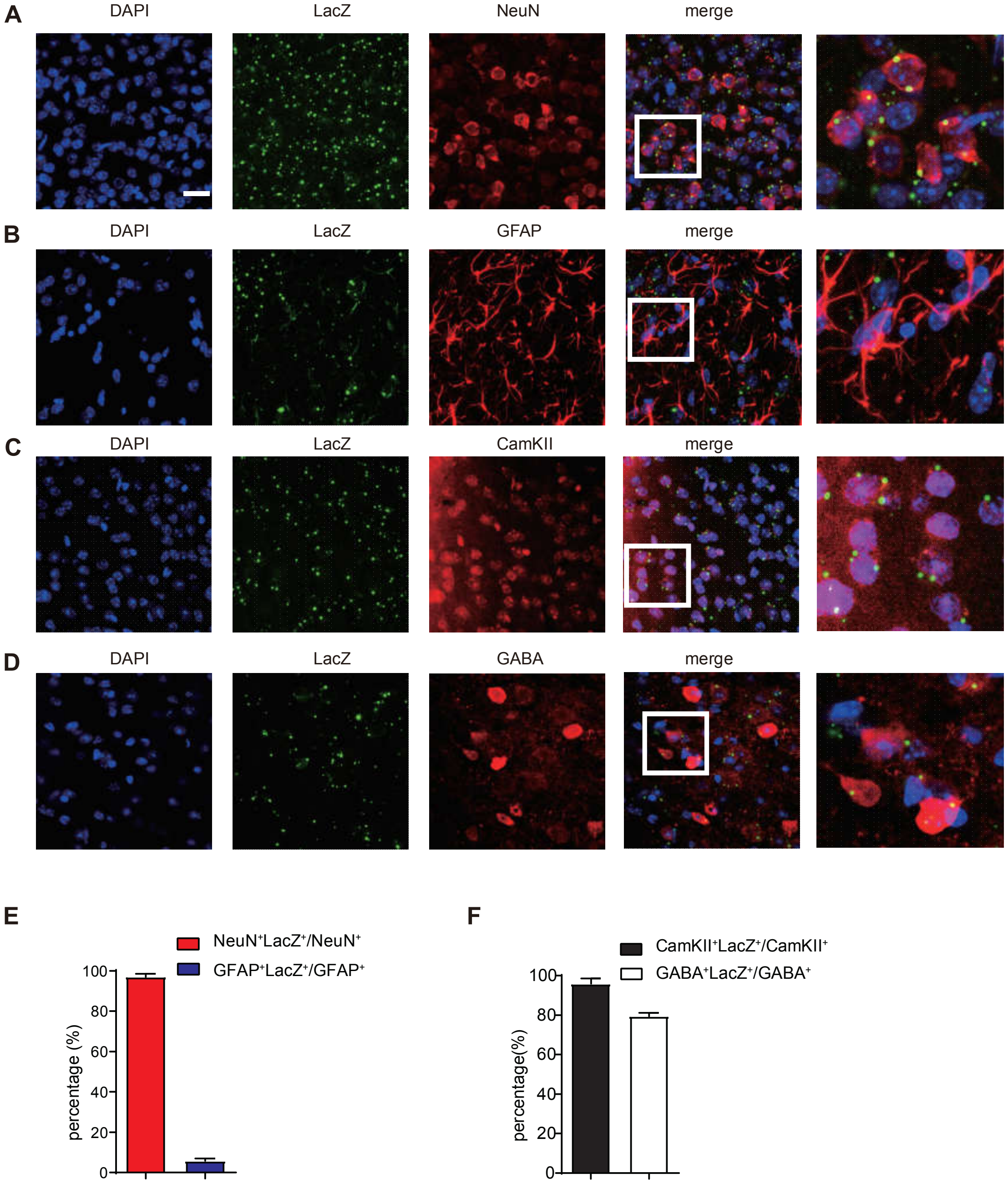

Supplementary Figure2

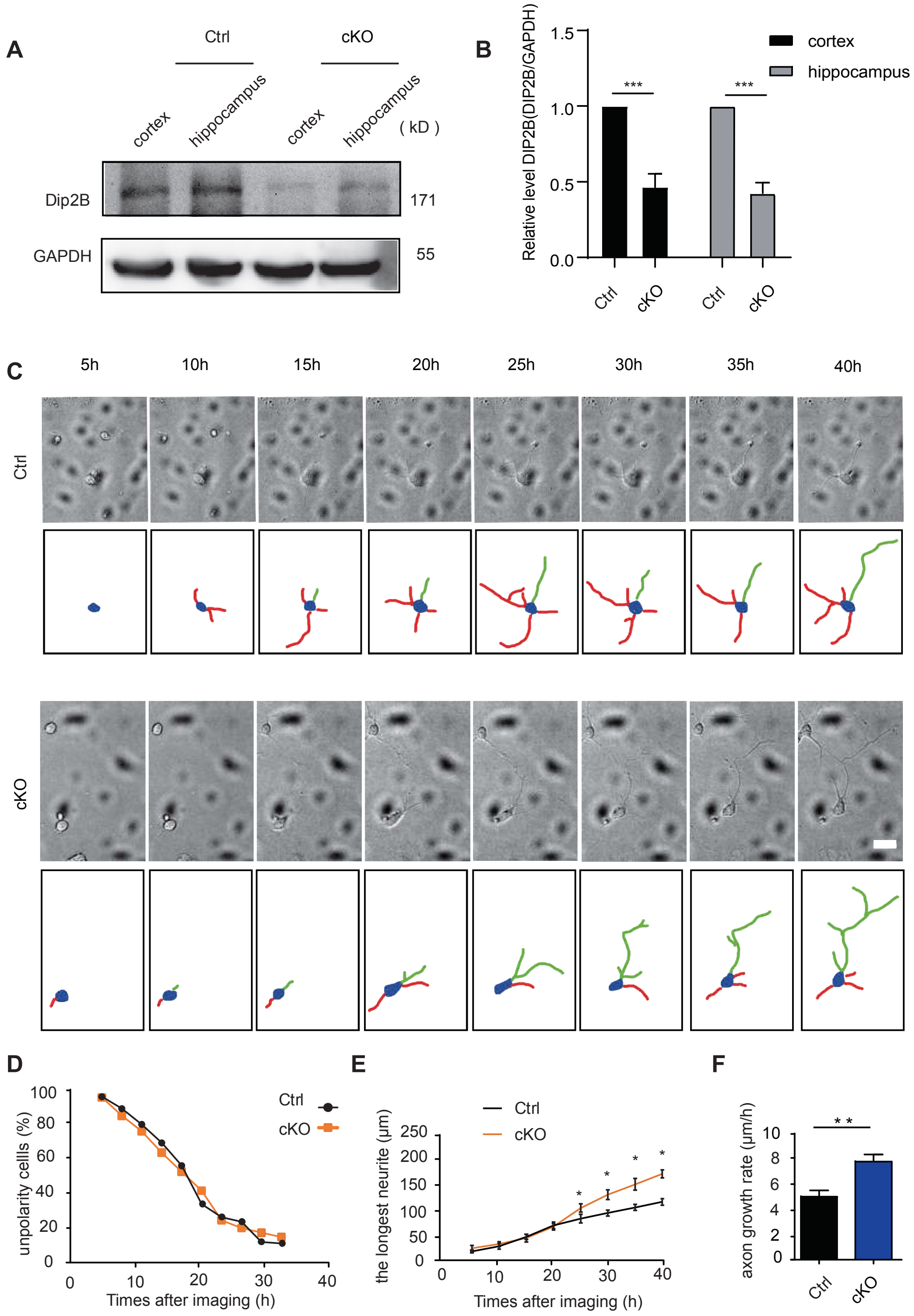

Supplementary Figure 3

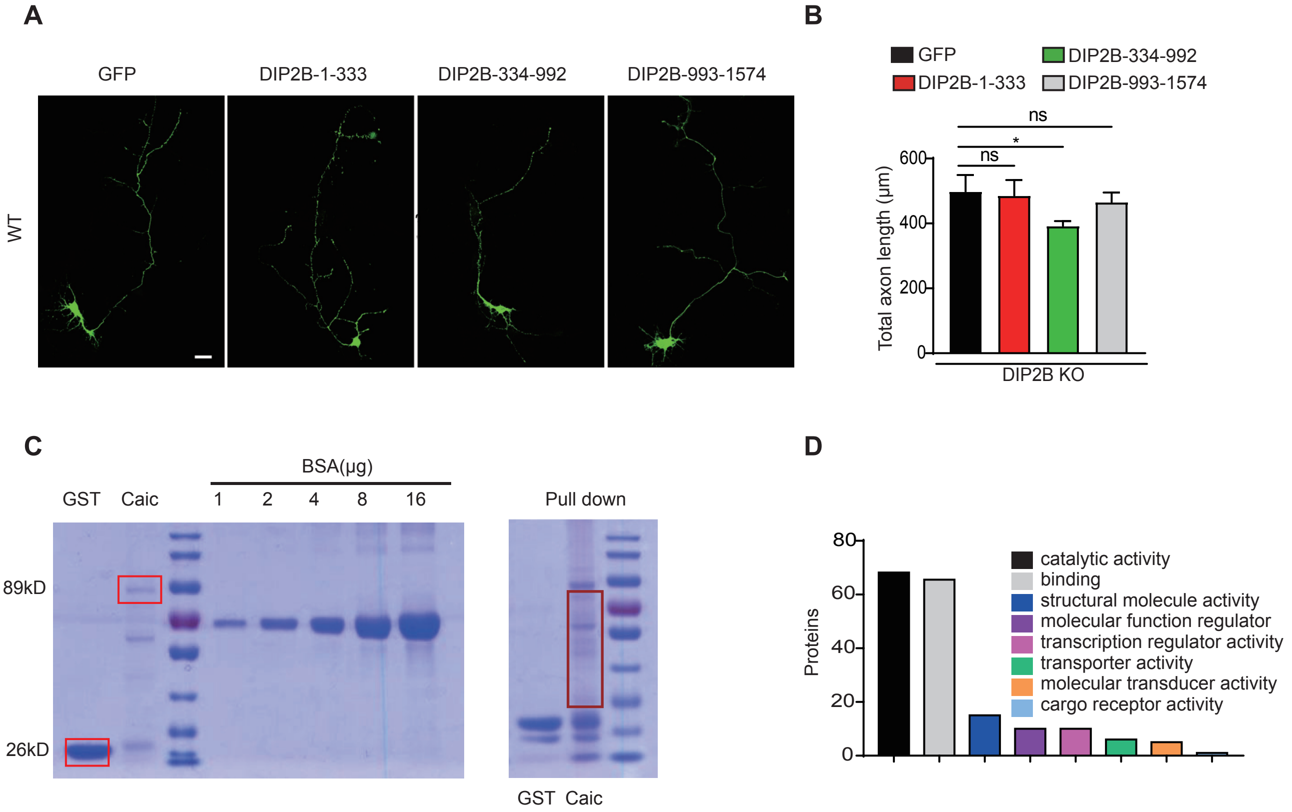

E Microtubule related proteins found from LC-MS/MS

Genes ID	Genes name	Protein ID	Protein Name
22143	Tuba1b	P05213	Tubulin alpha-1B chain
22145	Tuba4a	P68368	Tubulin alpha-4A chain
227163	Tubb4b	P68372	Tubulin beta-4B chain
140810	Ttbk2	A2AW15	Tau-tubulin kinase 2
72948	Tppp	Q3URG1	tubulin polymerization promotin protein
17756	Map2	P20357	Microtubule-associated protein 2
17762	Mapt	B1AQW2	Microtubule-associated protein tau
17754	Map1a	A2ARP8	Microtubule-associated protein 1A

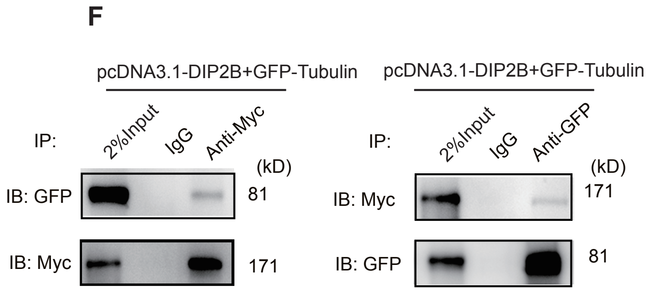

Supplementary Table1 Detail Data Analysis

Figure No.	Conditions	Average ± SEM	P value	F/t value
Fig. 2C	WT	471.6 ± 24.04	p < 0.001	t = 4.793
	KO	687.6 ± 35.15		
Fig. 2D	WT	255.3 ± 9.601	p < 0.001	t = 4.914
	KO	360.6 ± 18.13		
Fig. 2F	WT total	4.342 ± 0.2333	p < 0.001	t = 3.788
	KO total	6.294 ± 0.409		
	WT primary	2.474 ± 0.2377	p < 0.001	t = 3.262
	KO primary	3.863 ± 0.3218		
Fig. 2H	WT	72.49 ± 6.412	p = 0.0375	t = 2.098
	KO	55.54 ± 4.989		
Fig. 3A	scramble	1	scramble vs.shRNA3206: p=0.0073<br>scramble vs. shRNA3959: p=0.0025	F(2,6)=0.7126
	shRNA3206	0.4053 ± 0.02018		
	shRNA3959	0.2784 ± 0.02568		
Fig. 3C	scramble	445.2 ± 49.69	scramble vs.shRNA3206: p=0.0373<br>scramble vs. shRNA3959: p=0.0225	F (2, 44) = 0.788
	shRNA3206	639.9 ± 61.89		
	shRNA3959	679.6 ± 69.46		
Fig. 3D	scramble	72.49 ± 6.412	scramble vs.shRNA3206: p=0.0395<br>scramble vs. shRNA3959: p=0.0446	F (2, 233) = 0.1845
	shRNA3206	53.52 ± 5.555		
	shRNA3959	55.54 ± 4.989		
Fig. 3E	scramble	278.8 ± 21.22	scramble vs.shRNA3206: p=0.0180<br>scramble vs. shRNA3959: p=0.0114	F (2, 93) = 2.135
	shRNA3206	384.9 ± 26.79		
	shRNA3959	387.4 ± 32.17		
Fig. 3F	scramble total	4.553 ± 0.202	scramble vs.shRNA3206: p=0.0023<br>scramble vs. shRNA3959: p=0.0021	F (2, 137) =11.23
	shRNA3206 total	6.549 ± 0.3568		
	shRNA3959 total	6.294 ± 0.409		
	scramble primary	2.5 ± 0.1951	scramble vs.shRNA3206:p=0.006<br>scramble vs. shRNA3959:p=0.0023	F (2, 130) =7.41
	shRNA3206 primary	4.02 ± 0.2708		
	shRNA3959 primary	3.863 ± 0.3218		
Fig. 4D	25h WT	86.1 ± 4.540	25h WT vs. 25h KO: p=0.0228	t=2.376
	25h KO	106.1 ± 7.196		
	30h WT	90.37 ± 6.301	30h WT vs. 30h KO: p=0.0319	t=3.524
	30h KO	133.8 ± 11.81		
	35h WT	100.6 ± 5.937	35h WT vs. 35h KO: p=0.0247	t=4.566
	35h KO	160.7 ± 14.49		
	40h WT	113.4 ± 15.247	40h WT vs. 40h KO: p=0.0235	t=5.475
	40h KO	178.6 ± 5.153		
Fig. 4E	WT	4.434 ± 0.976	p < 0.001	t = 3.626
	KO	8.988 ± 0.7034		
Fig. 5B	WT	932.7 ± 59.82	p < 0.001	t = 10.22
	KO	222.5 ± 35.32		
Fig. 5D	WT	2.369 ± 0.1777	p = 0.0029	t = 3.306
	KO	1.549 ± 0.1728		
	WT	23.08 ± 1.437	p = 0.3318	t = 0.9898
	KO	21.36 ± 1.024		
Fig. 6C	KO+GFP	792.6 ± 77.65	KO + GFP vs. KO +DIP2B-1-333: p=0.9777,<br>KO + GFP vs. KO +DIP2B-334-992: p=0.0485,<br>KO + GFP vs. KO +DIP2B-993-1574: p=0.7375	F(3.80)=2.849
	KO+DIP2B-1-333	754.8 ± 57.31		
	KO+DIP2B-334-992	639.3 ± 38.00		
	KO+DIP2B-993-1574	722.5 ± 36.80		
Fig. 6G	WT	1.035 ± 0.1332	p = 0.0041	t=3.703
	KO	0.7583 ± 0.1255		
Fig. 6H	KO+GFP	787.3 ± 68.78	KO+GFP vs. KO+DIP2B-GFP: p<0.001,<br>KO+GFP vs. KO+tubulin : p=0.4151,<br>KO+GFP vs. KO+tubulin-K40Q: p=0.0454,<br>KO+tubulinvs. KO+tubulin-K40Q: p=0.0.0476	F (3, 63) =7.78
	KO+DIP2B	355.3 ± 29.25		
	KO+tubulin	787.4 ± 60.75		
	KO+K40Q	601.3 ± 32.82		
Fig. S2B	cortex Ctrl	1	p<0.001	t=12.21
	cortex cKO	0.4654 ± 0.04378		
	hippocampus Ctrl	1	p<0.001	t=16.83
	hippocampus cKO	0.4257 ± 0.03412		
Fig. S2E	25h Ctrl	90.1 ± 6.871	25h WT vs. 25h cKO: p=0.0368	t=3.254
	25h cKO	107.1 ± 7.145		
	30h Ctrl	100.37 ± 8.325	30h WT vs. 30h cKO: p=0.0254	t=4.687
	30h cKO	135.8 ± 10.678		
	35h Ctrl	110.6 ± 9.867	35h WT vs. 35h cKO: p=0.0435	t=6.354
	35h cKO	158.7 ± 13.579		
	40h Ctrl	120.4 ± 12.587	40h WT vs. 40h cKO: p=0.0371	t=5.879
	40h cKO	171.6 ± 4.658		
Fig. S2F	Ctrl	4.397 ± 0.8324	p<0.001	t = 3.562
	cKO	7.735 ± 0.7032		
Fig. S3B	WT+GFP	498.2 ± 50.80	WT+GFP vs. WT+DIP2B-1-333: p=0.9777,<br>WT+GFP vs. WT+DIP2B-334-992: p=0.0485,<br>WT+GFP vs. KO +DIP2B-993-1574: p=0.7375	F(3.80)=2.849
	WT+DIP2B-1-333	485.8 ± 47.84		
	WT+DIP2B-334-992	392.5 ± 30.68		
	WT+DIP2B-993-1574	466.5 ± 29.12		
